# Cognitive Impairment Before Cardiac Surgery: A Prospective Single-Center Observational Analysis

**DOI:** 10.3390/jcm14061853

**Published:** 2025-03-10

**Authors:** Juan M. Perdomo, Manuel López-Baamonde, Elena Gimeno-Santos, Ricard Navarro-Ripoll, María José Arguis, Antonio López-Hernández, Adrià Tort-Merino, Mircea Balasa, Raquel Sebio-Garcia, Eva Rivas, Graciela Martínez-Pallí

**Affiliations:** 1 Department of Anaesthesiology, Hospital Clínic de Barcelona, Villarroel 170, 08036 Barcelona, Spain; jmperdom@clinic.cat (J.M.P.);; 2 Physical Medicine and Rehabilitation Department, Hospital Clínic de Barcelona, Villarroel 170, 08036 Barcelona, Spain; 3 Institut d’Investigació Albert Pi i Sunye (IDIBPAS), University of Barcelona, Casanova 143, 08036 Barcelona, Spain; 4 Department of Neurology, Hospital Clínic de Barcelona, Villarroel 170, 08036 Barcelona, Spain; 5 CIBER Enfermedades Respiratorias (CIBERES), 28029 Madrid, Spain; 6 Outcomes Research Consortium, Houston, TX 77026, USA

**Keywords:** perioperative cognitive disorders, cardiac surgery, preoperative assessment, physical fitness

## Abstract

**Background/Objectives**: We aimed at evaluating the prevalence of cognitive impairment before cardiac surgery, its associated risk factors, and the diagnostic performance of cognitive tests. **Methods**: This prospective, single-center observational study included patients aged 50 years or older with coronary artery and/or valvular heart disease waiting for cardiac surgery. Patients underwent a cognitive and physical assessment before cardiac surgery. The cognitive assessment included eight tests exploring different cognitive domains and two questions exploring subjective cognitive complaints. Physical assessment included functional capacity and physical activity level. Cognitive tests with adjusted scores below 1.5 or more standard deviations from cognitively unimpaired subjects were considered abnormal. Cognitive impairment was defined as two or more abnormal cognitive tests. **Results**: We identified objective cognitive impairment in 41 out of 134 patients (31%). Interestingly, 66% of patients with objective cognitive impairment did not report any complaints. Moreover, similar complaints were reported among patients with and without objective cognitive impairment. The combination of Phonetic Fluency Test, Trail Making Test B, Digit Modalities Test, and the digit span forwards from the Wechsler Adult Intelligence Scale yielded the best diagnostic accuracy (AUC: 0.88; 95 CI: 0.82–0.93). Finally, cognitive impairment was associated with a worse Sit-To-Stand performance. **Conclusions**: Objective cognitive impairment before cardiac surgery is prevalent but subjective cognitive complaints are unreliable. We propose a combination of four cognitive tests with an efficient diagnostic profile to enhance its clinical applicability.

## 1. Introduction

Cognitive impairment before surgery is associated with an increased risk of subsequent cognitive deterioration after surgery, postoperative complications, prolonged hospital stay, and mortality [1,2]. While preoperative cognitive impairment prevalence in non-cardiac surgical patients ranges from 27% to 57%, it is particularly critical in cardiac surgery due to the higher risk of postoperative cognitive dysfunction in this population [3,4,5,6].

Even a subtle cognitive deterioration in previously impaired patients may have a huge impact on their quality of life and should be considered during the informed consent for surgery [6]. Although there is limited evidence, several perioperative strategies have been identified as “potentially protective” against postoperative cognitive decline [7]. Therefore, the preoperative assessment of the cognitive function represents a key opportunity to identify modifiable risk factors and physical conditions that surgical teams, patients, and relatives can address to achieve better outcomes. However, preoperative cognitive impairment is often under-recognized in daily practice [8]. This is likely due to the lack of consensus regarding optimal assessment tools during the preoperative period. While some advocate for a single screening test [4], several studies and expert recommendations have shown that using a single screening tool may be inadequate, as not all cognitive domains are uniformly affected [3,9,10].

Cognitive impairment before cardiac surgery has been reported to have a prevalence ranging from 8% to 35% according to different studies [11,12,13,14,15,16,17,18,19,20]. However, these studies were mainly focused on patients undergoing coronary artery bypass grafting (CABG)—likely due to their presumed higher risk attributable to the associated cardiovascular risk factors. Hence, a broader comprehensive preoperative cognitive assessment of all cardiac surgery patients (including both coronary and valvular procedures) may provide valuable insights into the extent, characteristics, and potentially associated risk factors of a poor cognitive status before surgery.

Therefore, we aimed to evaluate the prevalence of cognitive impairment among patients waiting for cardiac surgery, and to characterize the most affected cognitive domains. Additionally, we sought to identify the risk factors independently associated with cognitive impairment and to analyze the best diagnostic performance out of a combination of cognitive tests.

## 2. Materials and Methods

This is a sub-study of a prospective, single-center, open-label, randomized, controlled clinical trial (Registered at ClinicalTrials.gov; NCT03466606). The primary outcomes of this sub-study were established a priori at the initiation of the study. This manuscript adheres to the applicable STROBE statement. The aim of the main trial was to evaluate the impact of a multimodal prehabilitation program on postoperative complications among patients undergoing cardiac surgery, compared to routine preoperative care [21]. Approval for this study was obtained from the Ethics Committee of the Hospital Clinic of Barcelona on 3 May 2018 (HCB/2017/0708). The inclusion criteria were age ≥ 50 years, planned for elective CABG and/or heart valve surgery, expected waiting time for surgery ≥ 6 weeks, and written informed consent. Patients with physical limitations preventing physical assessments or participation in the prehabilitation program, reading difficulties impeding full comprehension of the study, and non-Spanish or Catalan native speakers were excluded. Patients with logistic constraints hindering their attendance to the supervised training sessions at the hospital were also excluded.

We collected the participants’ morphometric characteristics, comorbidities, cardiovascular risk factors, chronic medications, tobacco and alcohol consumption habits, and years of education. We used The European System for Cardiac Operative Risk Evaluation (EuroSCORE II) to calculate the surgical risk of the patients [22]. We also assessed the level of frailty with the clinical frailty scale from the Canadian Study of Health and Aging [23], and the emotional status with the Hospital and Anxiety Depression Scale (HADS) [24].

### 2.1. Cognitive Assessment

We selected a battery of tests with input from a neurologist and a neuropsychologist—both experts on cognitive disorders. Two blinded trained clinicians (JP, ML), who were comprehensibly trained by the neuropsychologist to follow the specific administration instructions of each test, performed the cognitive assessment. One of the two trained clinicians performed each cognitive evaluation entirely in a private room without the patient’s relatives. A briefing was conducted before the cognitive assessment to explain the logistic aspects of the tests. All tests were paper-based and performed in the same order, consecutively, and without interruptions (Table 1) [25,26,27,28,29,30]. In the Memory Alteration Test, participants were asked to repeat and recall predefined worlds and sentences, as well as answer specific questions. During the digit forwards and backwards subtests, participants were asked to repeat an increasing number of digits. During the Trail Making Test A, participants were asked to connect numbers from 1 to 25, and numbers and letters for the Trail Making Test B, randomly arranged, as quickly as possible. During the Symbol Digit Modalities Test, based on a key consisting of a series of symbols paired with digits, participants were asked to fill in the blanks on the test sheet with the correct digit under each symbol, as quickly as possible. During the Semantic Fluency Test, participants verbalized as many animals as possible within one minute. During the Phonetic Fluency Test, participants verbalized as many words as possible beginning with P, M, and R. The time required to perform all tests was between 25 and 45 min. We adjusted raw scores to age and years of education for each of the 8 tests (excluding MMSE) to obtain the scaled scores using validated Spanish normative data from cognitively unimpaired controls [31]. Based on previous recommendations, tests with a decline of 1.5 or more standard deviation, on scaled scores against norms, were defined as abnormal [9,31]. The primary outcome was objective cognitive impairment, defined as two or more abnormal cognitive tests, excluding MMSE [5,9,14,20,32]. Additionally, after the test completion, patients were queried regarding subjective cognitive complaints by asking “Have you perceived any cognitive problems recently?” and “Have you experienced any memory problems recently?”. If needed, we explained the phrase “cognitive problems” as any difficulties concentrating, understanding sentences, or following simple instructions. We defined a cognitive complaint as a positive answer to any of these two questions.

### 2.2. Physical Assessment

Physical assessments included the New York Heart Association (NYHA) functional classification [33], the functional capacity of performing an incremental cardiopulmonary exercise test (CPET) [34], the endurance time during a cycling constant work-rate exercise test at a load equivalent to 80% of the peak workload the patient managed to tolerate on the CPET (Ergoline 900, Ergoline, Bitz, and Ergocard Professional, Medisoft; Sorinnes, Belgium), the distance covered in the 6-minute walking test (6MWT) [35], the hand grip strength test (Jamar Hydraulic Hand Dynamometer; Sammons Preston; Bolingbrook, IL, USA) [36], and the 30 s Sit-To-Stand (STS) test [37]. Functional capacity was estimated using the Duke Activity Status Index (DASI) questionnaire [38]. Physical activity levels were determined by the Yale Physical Activity Survey (YPAS) [39].

### 2.3. Statistical Analysis

We used the Shapiro–Wilk test to determine data distribution [40]. Categorical data were expressed as n (%), and continuous data either as mean and standard deviation (SD) or as median and interquartile range (IQR), as appropriate. We performed univariate analyses to compare the patients with and without cognitive impairment using the Chi-square test for categorical variables and Student’s *t* test for continuous variables (or a Mann–Whitney test if continuous variables were non-normally distributed). The strength of association, when present, was measured in terms of odds ratio (OR), with statistical significance set at alpha value of 5% (two-tailed).

In a post hoc analysis, we evaluated the diagnostic performance of different test combinations to identify cognitive impairment. Each combination of two tests underwent an evaluation based on its area under the curve (AUC) in the receiver operating characteristic (ROC) curve analysis. Initially, all tests were assessed, and the one with the highest AUC was selected. Subsequently, this chosen test was paired with each of the other tests, and each combination was again re-evaluated for AUC. The combination exhibiting the highest AUC was then merged with the remaining tests in subsequent iterations. This iterative process continued, incorporating the highest AUC, until no further increase in AUC was observed. The resulting AUC from each iteration was plotted. Analysis was performed with R version 3.6.1 using RStudio version 2023.12.1 (Posit, Boston, MA, USA).

## 3. Results

**Figure 1:**
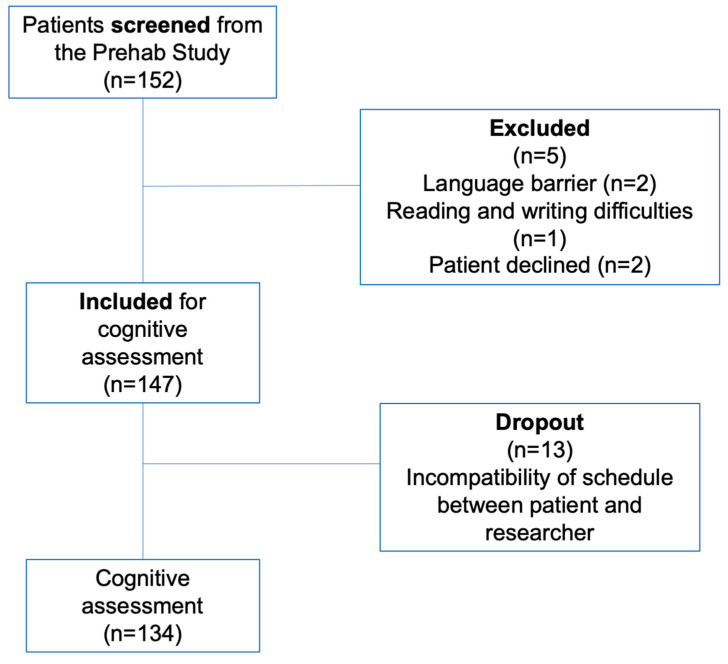
Study flowchart.

We approached a total of 152 patients for potential participation, and 147 patients consented to participate (from 29 May 2018 and 3 May 2021). Ultimately, 13 patients were excluded due to scheduling conflicts between the researcher and the participants. Consequently, 134 participants completed the cognitive assessment (Figure 1). Patients’ characteristics, frailty, and HADS scores are presented in Table 2. Table 3 presents the main variables of the physical assessment.

**Figure 2:**
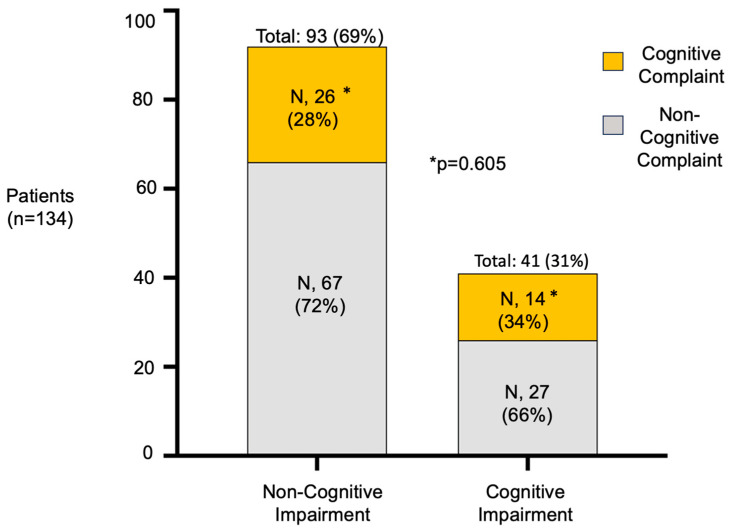
Distribution of cognitive complaints among patients with and without cognitive impairment. Cognitive impairment defined as two or more tests, excluding MMSE, scoring 1.5 standard deviation below normative expectations.

**Figure 3:**
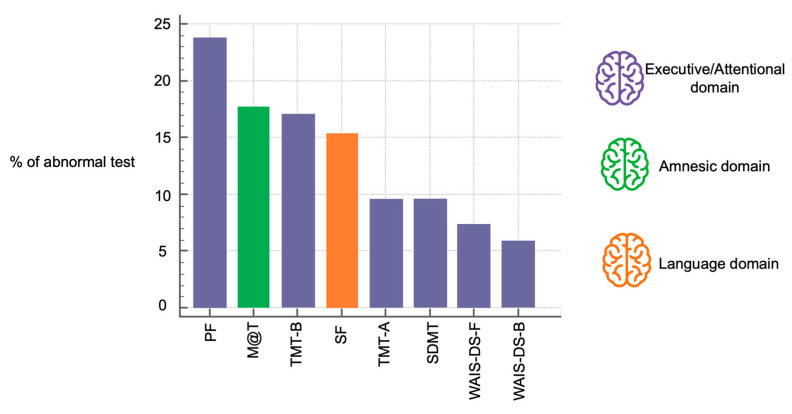
Percentage of patients scoring 1.5 standard deviation below normative expectations across the eight cognitive tests. Tests in purple asses executive/attentional functions, test in green assesses memory, and test in orange assesses language. M@T, Memory Alteration Test; WAIS-DS-F, WAIS Digit Spain Forwards; WAIS-DS-B, WAIS Digit Span Backwards; TMT-A, Trail Making Test A; TMT-B, Trail Making Test B; SDMT, Symbol Digit Modalities Tests; SF, Semantic Fluency; PF, Phonetic Fluency.

**Figure 4:**
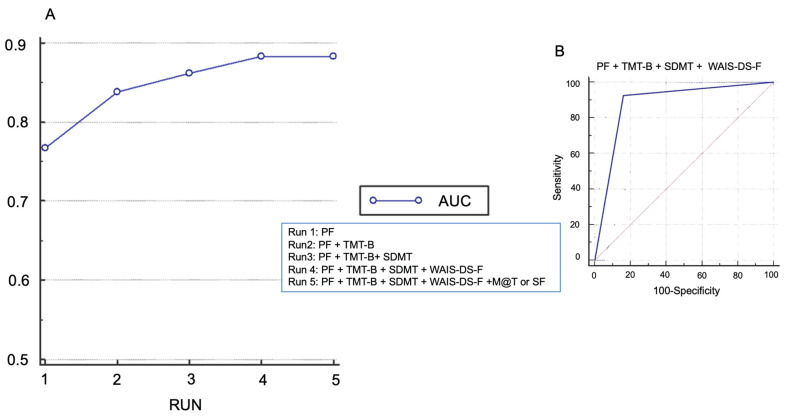
Combination of tests with the best diagnostic accuracy. (**A**). Different combination of tests until reaching the widest AUC at RUN 4. Notice that adding a fifth test does not increase the AUC at Run 5. (**B**). ROC curve of the best combination of tests. Continuous blue line for RUN 4. Orange line is the reference value for AUC 0.5. AUC, Area under curve; PF, Phonetic Fluency; TMT B, Trail Making Test B; SDMT, Symbol Digit Modalities Tests; WAIS-DS-F, WAIS Digit Span Forwards; M@T, Memory Alteration Test; SF, Semantic Fluency.

We identified objective cognitive impairment in 41 patients (31%). Notably, most patients with objective cognitive impairment (66%) did not report any cognitive complaint. (Figure 2) There was no difference in the prevalence of subjective cognitive complaints in patients with and without objective cognitive impairment (34% versus 28%, respectively, *p* > 0.05) (Figure 2). Executive and attention functions were the most affected cognitive domains (in 44% of participants), followed by alterations in amnesic profile (18%) and the language area (16%) (Figure 3). The combination of the Phonetic Fluency Test, Trail Making Test B, SDMT, and the WAIS digit span (forwards) yielded the highest AUC [0.88; 95% CI (0.82, 0.93)], with sensitivity of 93% and specificity of 84% (Figure 4). Adding a fifth test did not improve the AUC (Figure 4). The MMSE showed an AUC of 0.76, with sensitivity of 34% and specificity of 99%.

We found no association between objective cognitive impairment and participants’ age, years of education, Charlson comorbidity index, frailty, nor with the HADS. However, a significant association was observed with previous medical history of stroke [OR 9.36, 95% CI (1.854, 47.34)] (Table 2).

There were no differences in the prevalence of pre-existing cognitive impairment between patients having CABG or valve surgery. We found no significant associations with the cardiac surgery risk score EuroSCORE II. However, we did observe an association with the presence of aortic regurgitation [OR 3.140, 95% CI (1.14, 8.69)], and preoperative left ventricular ejection fraction (LVEF) [median LVEF in patients with and without cognitive impairment was 60% vs. 56%, [OR 0.942, 95% CI (0.893, 0.995)]] (Table 2).

We successfully completed the functional assessment in all patients, except for the CPET in 58 patients due to COVID-19 restrictions. Overall, there was no significant association between the prevalence of cognitive impairment and the level of physical activity (YPAS) or other parameters of physical fitness (6MWT, DASI, and endurance time). Notably, the STS test was the only parameter significantly associated with cognitive impairment. Specifically, for each additional sit-to-stand repetition performed by a patient in 30 s, the odds of having preoperative cognitive impairment decreased by 13.6%, (Table 3).

When analyzing each cognitive domain separately, we observed that the abnormal executive and attention areas were associated with a low preoperative functional status using NYHA functional classification (*p* = 0.048) and aortic regurgitation (*p* = 0.019). The abnormal amnesic profile was more prevalent in patients older than 60 years (*p* = 0.008) and was also associated with history of tobacco use (*p* = 0.004), as well as low preoperative functional status using NYHA functional classification (*p* < 0.01). Among the 76 patients who performed the CPET, those with an abnormal amnesic profile had worse endurance time compared to those with a normal amnesic profile (226 versus 296 s, *p* = 0.04). No factors were associated with abnormalities in tests exploring language cognitive functions.

## 4. Discussion

We found objective cognitive impairment in one out of three patients. Surprisingly, subjective cognitive complaints were reported similarly by patients with and without objective cognitive impairment. Additionally, we observed that a combination of four tests improved the diagnosis accuracy, and that potentially modifiable preoperative factors such as functional capacity assessed by STS or endurance time were associated with abnormal cognitive tests.

Our study showed a similar prevalence to previous studies also using a complete battery of cognitive tests in patients waiting for CABG [20] and non-cardiac surgery [5]. Importantly, we observed no differences between the patients scheduled for CABG or valve surgery. These findings may challenge the prevailing notion that patients awaiting CABG, presumed to have more cardiovascular risk factors, are inherently more vulnerable to perioperative cognitive disorders than those waiting for valvular heart surgery or non-cardiac surgery [14,15,19,20].

Some recommendations for the definition of perioperative cognitive disorders, including cognitive impairment before surgery, emphasize the importance of subjective complaints and their impact on daily life activities [9]. In our study, only one third of patients with objective cognitive impairment reported subjective complaints. A previous study in non-cardiac surgery patients reported a 64% prevalence of cognitive complaints and a significant relationship with lower scores using the Mini-Cog [4]. However, the reliance on a single cognitive screening tool may limit the scope of the assessment. Subjective reports of cognitive difficulties do not always align well with objective findings, and assessing preoperative subjective cognitive complaints may have inherent limitations. First, subjective cognitive complaints may be over-reported due to stress and anxiety during the perioperative period and the cognitive assessment itself [41]. Second, patients with pre-existing cognitive impairment may lack insight into their cognitive state and thus under-report complaints [42]. Clearly, cognitive impairment is often not perceived by patients themselves, and clinicians should therefore actively screen for it, particularly in high-risk surgical patients.

The definition of objective cognitive impairment is based on the results of validated psychometric tests and varies across the previous studies [43]. The recommendations from experts on perioperative cognitive disorders define mild objective impairment when the results are 1–2 standard deviations below norms or controls. In contrast, objective impairment is considered major when the results are >2 standard deviations below norms or controls [9]. As in previous large clinical trials on cardiac surgery patients [20], we did not distinguish between mild or major cognitive impairment.

Moreover, it is strongly recommended that the diagnosis of cognitive impairment relies on validated psychometric tests rather than the exclusive use of cognitive screening tools that could seem simpler and more time-efficient, such as MMSE, Montreal Cognitive Assessment test or Mini-Cog [3,9]. Our findings, identifying a low MMSE sensitivity of only 34%, confirm that screening tools can easily miss patients with cognitive impairment. However, employing large batteries of cognitive tests may be time-consuming, induce mental fatigue, and become unfeasible [44]. Our cognitive assessment with the eight tests, MMSE, and questions about subjective complaints was completed within a maximum of 45 min. Previously, studies reported completing 13 tests in 1 h [18]. Different approaches are used in previous studies, employing fewer [14,19,20] or more [11,12,18] tests to diagnose cognitive impairment. Most studies focused on tests exploring the executive function, as evidence suggests that it is the most affected area [10]. Indeed, upon a detailed analysis, we found the executive domain to be the most affected, followed by memory and language. We suggest employing four tests (Phonetic Fluency, Trail Making Test B, SDMT, and the WAIS digit span—forwards), all assessing executive function, as the combination with the highest AUC, and the best sensitivity (93%) and specificity (84%). Interestingly, adding a fifth test to this combination did not improve diagnostic accuracy, suggesting that test selection is more critical than test quantity for effective detection of preoperative cognitive impairment.

Efforts to identify modifiable risk factors for cognitive impairment before cardiac surgery are crucial for developing strategies for the improvement of post-operative cognitive outcomes [6,7]. Our data show an exploratory association between cognitive impairment and non-modifiable factors, such as LVEF, and the presence of aortic regurgitation. However, only 18 patients had aortic regurgitation and the difference in the median LVEF (56% vs. 60%), albeit statistically significant, may be influenced by echocardiography operator-dependency [45]. Several pathways could explain the association between cardiac disease and cognitive impairment: firstly, a widened pulse pressure present in patients with aortic regurgitation may induce a proinflammatory brain state leading to worse cognitive performance [46]. Secondly, heart failure is characterized by an imbalance between the cardiac output and metabolic demands, resulting in reduced cerebral blood flow, which is linked to changes in the brain [47]. Finally, previous studies suggest that ischemic heart disease is associated with atherosclerotic disease that also affects cerebral perfusion, increasing the risk of cognitive dysfunction [48].

We also found that cognitive impairment was associated with modifiable factors related to the patients’ physical status. Specifically, poorer performance on the 30 STS was significantly associated with cognitive impairment, probably reflecting the interplay between muscle endurance and executive cognitive functions required for planning, coordinating, and executing low extremity tasks [49]. This finding may also be explained by the relationship between muscle strength, working memory, and information processing speed [50]. There is a well described neuromuscular weakness and poor balance present in patients with dementia, due to neuronal and dendritic loss, reduced branching, and cerebral hypoperfusion [51]. Better endurance time (cardiopulmonary endurance) on the CPET was associated with a better amnesic profile, and the patients with an abnormal executive and amnesic profile had a worse functional status on the NYHA. These findings suggest that perhaps the type of heart disease (coronary versus valvular) is less important than the impact of the heart disease on patients’ physical status. This aligns with the established links between the different components of physical fitness (muscle strength, muscle endurance, agility, flexibility, balance, and cardiopulmonary endurance) and cognitive function, particularly in older adults [52]. Although evidence is still scarce, prehabilitation, a preventive preoperative strategy targeting modifiable risk factors and involving physical activity, nutritional optimization, and psychological support has been previously proposed to improve preoperative cognitive reserve and prevent or minimize postoperative cognitive impairment [53,54,55].

Our study suffers from several limitations. Firstly, we included a relatively small sample size, which was not specifically calculated for the main outcome, thereby restricting our ability to draw definitive conclusions. Moreover, although the inclusion criteria did not specifically favor patients with valvular heart disease over coronary artery disease, around 80% of our patients underwent valve repair surgery. The main reason for this imbalance is that we are a hospital with an expert surgical team in mitral valve repair and a significant percentage of GABG surgery is performed as emergency cases (30%) or as preferent cases (60%), without the minimum 6 weeks expected waiting time for surgery needed for the inclusion criteria. This potential selection bias may impact other characteristics of the patients included in this study, such as age. We included younger patients since patients with mitral regurgitation are usually younger than those undergoing CABG; and we have a more homogenous age distribution in our sample [IQR: 64–77] that may mitigate the effect of age on cognition and prevent us from finding the association between age and pre-existing cognitive impairment previously described [14,15,20]. Despite this limitation, we were able to identify several significant associations. Secondly, our sample only included patients scheduled for valvular heart and/or CABG surgery, without representation of patients undergoing aortic surgery. Given the intricate nature of a cognitive assessment, we prioritized data quality over generalizability, so future research with larger sample sizes will be necessary to validate our findings and enhance the robustness of our conclusions. Although, we only considered a single composite primary outcome, we also analyzed several associations between baseline variables and specific domains and tests that comprised the composite outcome. These analyses were not corrected for multiple testing and should therefore be considered as exploratory. Finally, we did not assess the impact of preoperative cognitive dysfunction on the postoperative outcomes. In a metanalysis, the presence of cognitive impairment before cardiac surgery was associated with delirium, and ICU and hospital length of stay. However, none of the studies included in the metanalysis performed the cognitive assessment with a complete battery of cognitive tests and the GRADE evaluation of the postoperative outcomes, and involved very low-to-low-quality evidence [6].

The main strength of our study is the extensive and reproducible preoperative cognitive and functional assessment. Our detailed dataset allows us to conclude which combination of tests is the most accurate in assessing cognitive impairment, thereby enhancing feasible implementation in clinical settings.

Future research is needed to understand the implications of preoperative cognitive impairment over the perioperative cognitive function and postoperative outcomes in more depth. Imaging studies assessing the brain areas involved in this impairment will also help to better design the targeted therapies. Finally, we need robust evidence assessing the impact of prehabilitation strategies on the preoperative cognitive reserve and whether it can prevent or minimize postoperative cognitive impairment.

## 5. Conclusions

In conclusion, our main result demonstrates that objective cognitive impairment affects nearly a third of patients waiting for cardiac surgery who are often unaware of their condition. This result emphasizes the critical need for routine preoperative cognitive function assessment. We have identified a combination of four cognitive tests with an efficient diagnostic profile, streamlining the time required for a cognitive assessment and enhancing clinical applicability. Additionally, we found that a poorer performance in the 30 s STS is associated with cognitive impairment, highlighting it as a potentially modifiable risk factor and a target for intervention.

## Figures and Tables

**Table 1:** Cognitive tests, the order the were performed, and clinical applicability.

Order	Test	Validated Use
1	Memory Alteration Test (M@T)	Screening of mild amnestic cognitive impairment and early Alzheimer’s disease
	Wechsler Adult Intelligence Scale (WAIS-III)	
2	Digits forwards subtest	To explore attention span
3	Digits backwards subtest	To explore working memory
4	Trail Making Test A	To explore visual-motor skills, attention, sequencing, and cognitive flexibility
5	Trail Making Test B	
6	Symbol Digit Modalities Test (SDMT)	to explore cognitive processing speed
7	Semantic Fluency Test	To explore language domain
8	Phonetic Fluency Test	To explore executive function domains
9	Mini-Mental State Examination (MMSE)	Global cognitive screening tool

**Table 2:** Patients’ characteristics and association with cognitive impairment. IQR, Interquartile Range; CSHA, Canadian Study of Health and Aging; HADS, Hospital and Anxiety Depression Scale; LVEF, Left Ventricular Ejection Fraction; OR, Odds Ratio; 95% CI, 95% Confidence Interval.

Characteristics	Total	Cognitive Impairment		Association with Cognitive Impairment	
	(*n* = 134)	Yes (*n* = 41)	No (*n* = 93)	OR (95% CI)	*p* Value
Age (yr) [IQR]	70 [63–77]	74 [64–79]	69 [63–79]	1.039 (0.993, 1.088)	0.06
Sex				1.078(0.461–2.525)	0.862
Female (%)	34 (25)	10 (24)	24 (26)		
Male (%)	100 (75)	31 (76)	68 (74)		
Ever smoker (%)	81 (60)	22 (54)	59 (63)	0.667 (0.317, 1.405)	0.339
Previous stroke (%)	9 (7)	7 (17)	2 (2)	9.368 (1.854, 47.34)	0.004
Charlson [IQR]	3 [3–5]	3 [2–5]	4 [3–5]	0.984 (0.815, 1.188)	0.87
Vulnerable and mildly frail patients (CSHA > 3) (%)	78 (58)	22 (58)	49 (55)	1.227 (0.565, 2.668)	0.70
HADS [IQR]	6 [4–11]	6 [4–14]	7 [4–10]	1.041 (0.979, 1.107)	0.17
Years of education [IQR]	12 [9–19]	12 [8–20]	12 [10–19]	0.990 (0.950–1.031)	0.46
Type of Heart Disease (%)				1.349 (0.875, 2.17)	0.17
Isolated Valvular Heart Disease	92 (69)	25 (61)	67 (72)		
Isolated Coronary Heart Disease	18 (13)	6 (15)	12 (13)		
Combined surgery	24 (18)	10 (24)	14 (15)		
Mitral Stenosis	8 (6)	0 (0)	8 (9)	0.914 (0.859, 0.973)	0.11
Mitral Regurgitation	42 (34)	14 (34)	28 (30)	1.204 (0.550, 2.634)	0.69
Aortic Stenosis	53 (40)	14 (34)	39 (42)	0.718 (0.334, 1.544)	0.45
Aortic Regurgitation	18 (13)	9 (22)	9 (10)	3.140 (1.14, 8.69)	0.04
Tricuspid valve Disease	3 (2)	0 (0)	3 (3)	0.967 (0.932, 1.004)	0.55
Median LVEF [IQR]	60 [55–64]	56 [55–60]	60 [55–65]	0.942 (0.893, 0.995)	0.03
Median EuroSCORE II [IQR]	1.3 [0.9–2.3]	1.4 [0.8–2.3]	1.3 [1.0–2.4]	0.942 (0.761, 1.167)	0.56

**Table 3:** Physical assessment and association with cognitive impairment. Values are presented in means and medians. IQR, Interquartile Range; SD, Standard Deviation; YPAS, Yale Physical Activity Survey; DASI, Duke Activity Status Index; CPET, cardiopulmonary exercise test; NYHA, New York Heart Association functional classification.

Characteristics	Total	Cognitive Impairment		Association with Cognitive Impairment	
	(*n* = 134)	Yes (*n* = 41)	No (*n* = 93)	OR [95% CI]	*p* Value
YPAS Summary index of physical activity [IQR]	38 [27–48]	37 [27–46]	38 [26–51]	0.997 (0.977, 1.018)	0.79
DASI [IQR]	28 [19–41]	26 [16–43]	29 [22–40]	0.981 (0.954, 1.01)	0.19
6-Minute Walking Test—Final distance (m) [SD]	475 [89]	476 [394–536]	484 [438–540]	0.997 (0.993, 1.001)	0.14
CPET- Endurance Time (s) [IQR] n = 76	282 [219–360]	278 [213–364]	291 [230–355]	0.999 (0.995, 1.003)	0.61
NYHA 3–4 (%)	22 (16)	9 (24)	13 (14)	1.886 (0.729, 4.879)	0.37
Sit to Stand Test—Number of repetitions [IQR]	11 [9–14]	10 [9–13]	12 [10–14]	0.864 (0.767, 0.972)	0.02
Hand Grip Dominant Strength (kg) [SD]	32 [10]	28 [22–36]	32 [26–39]	0.985 (0.948, 1.023)	0.43

## Data Availability

The data supporting this study is presented in the manuscript. Further data are available on request from the corresponding author.
